# When fur and feather occur together: interclass transmission of avian influenza A virus from mammals to birds through common resources

**DOI:** 10.1038/srep14354

**Published:** 2015-09-24

**Authors:** J. Jeffrey Root, Susan A. Shriner, Jeremy W. Ellis, Kaci K. VanDalen, Heather J. Sullivan, Alan B. Franklin

**Affiliations:** 1 United States Department of Agriculture, National Wildlife Research Center, Fort Collins, CO, USA

## Abstract

The potential role of wild mammals in avian influenza A virus (IAV) transmission cycles has received some attention in recent years and cases where birds have transmitted IAV to mammals have been documented. However, the contrasting cycle, wherein a mammal could transmit an avian IAV to birds, has been largely overlooked. We experimentally tested the abilities of two mammalian species to transmit avian IAV to mallards (*Anas platyrhynchos*) in simulated natural environments. Results suggested that striped skunks (*Mephitis mephitis*) can successfully transmit avian IAV to mallards through indirect contact with shared resources, as transmission was noted in 1 of 4 of the mallards tested. Cottontail rabbits (*Sylvilagus* sp.) exhibited a similar pattern, as one of five cottontail rabbits successfully transmitted IAV to a mallard, likely through environmental contamination. For each mammalian species tested, the mallards that became infected were those paired with the individual mammals with the lowest shedding levels but were anecdotally observed to be the most active animals. Mammals associated with and around poultry rearing facilities should be taken into consideration in biosecurity plans.

Avian influenza A viruses (IAV) are primarily hosted by birds in the orders Anseriformes and Charadriiformes (i.e., waterfowl and shorebirds)^1^. On a local scale, contaminated water leading to fecal-oral transmission is thought to be an important transmission route in waterfowl populations in aquatic habitats^2^. Regionally, the movement of IAVs can occur rapidly along migratory routes, such as the Mississippi flyway^3^. Globally, migrating birds have been suggested as a potential mechanism for the intercontinental spread of an H5N8 virus^4^.

Select mammalian species have been recently evaluated for their potential roles in influenza A virus ecology^5^,^6^,^7^,^8^. While it has been shown that avian species can transmit IAVs to mammals in certain situations^8^,^9^, the opposite scenario, in which a mammal might transmit an IAV to birds through direct contact or shared resources, has not been thoroughly evaluated. Considering that some avian species, such as mallards (*Anas platyrhynchos*), are highly susceptible to avian IAVs, this scenario, when coupled with the oral route of infection, might actually be quite plausible at local scales when mammals and birds share resources.

The past several years have yielded the discovery of several novel IAVs. While some, such as an Asian-origin H7N9 virus have caused major public health threats^10^, others, such as highly pathogenic (HP) H5N8 have infected a large number of poultry farms^11^. Furthermore, a novel HP IAV H5N8 and HP reassortants of this virus have been recently introduced into and/or detected in North American bird populations^12^. These novel and emerging viruses reaffirm the need to assess multiple facets of farm-scale biosecurity of poultry operations, wherein all routes of viral intrusion, even those thought to be unlikely, are addressed.

The existence of mammalian wildlife on poultry operation facilities has been proposed to potentially influence risks associated with the diffusion of low pathogenic (LP) IAV among different facilities^13^. Striped skunks (*Mephitis mephitis*) and cottontail rabbits (*Sylvilagus* sp.) are two ubiquitous peridomestic mammalian species that are known to frequent and live in and near farmsteads. Of interest, both of these species have been shown to successfully replicate avian IAV^6^,^14^. For these reasons, the objective of this study was to assess the capacity of select peridomestic mammals infected with IAV to transmit the virus indirectly to mallards through shared resources or directly/indirectly through shared pens.

## Methods

### Overview

Two experiments were conducted to test if striped skunks and cottontail rabbits can transmit LP IAV to mallards. In experiment 1, striped skunks were paired with mallards and “mirrored” pens (i.e., outfitted with the same items) were exchanged to determine if mallards can become infected through environmental contamination. In experiment 2, cottontail rabbits and mallards were briefly co-housed and subsequently switched pens to assess if mallards can become infected through direct or indirect contact. The institutional animal care and use committee of the National Wildlife Research Center approved the experiments which were performed in accordance with relevant guidelines.

### Study animals

**Figure 1:**
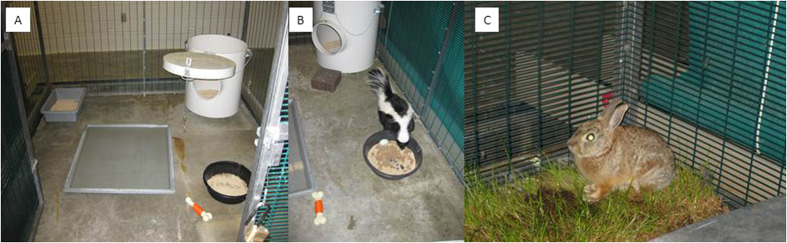
(**A**) shows the experimental configuration of striped skunk pens that included a den box, a litter box, a shallow pond, a mixed food bowl, and an enrichment toy. A second watering device was added to the pen at 1 day post infection. (**B**) shows a striped skunk utilizing a food dish containing duck and skunk feed. (**C**) shows a cottontail rabbit utilizing the sod used in the cottontail-mallard experiment. The cottontail-mallard experimental configuration had several unique attributes when compared to the setup of the striped skunk-mallard configuration (see Methods).

Four striped skunks (purchased from a commercial vendor) were utilized in the first experiment. Prior to the initiation of experimentation, a blood sample was taken from each skunk to test for antibodies reactive with IAV. On day 0 of the experiment, the skunks were moved into animal biosafety level-2 (ABSL-2) facilities and housed in 7′w × 8′d × 7′ h pens. All animals were supplied with a den box, a shallow pond, a food bowl, a litter box, an enrichment toy, and a poultry watering device (placed into the pen on day 1; Fig. 1). Food (Omnivore Diet; Mazuri, Purina Mills, LLC, St. Louis, MO; Chick Starter; Ranch-Way Feeds, Fort Collins, CO; both feeds placed in the same food tray) and water were replenished each day when needed. Water treated to remove chlorine and other chemicals was used throughout the experiments for both the ponds and the watering devices.

Five cottontail rabbits (*Sylvilagus* sp.) were live captured in Larimer County, CO. These animals were held in quarantine for >14 days during which a blood sample was taken from them to test for antibodies reactive with influenza A virus. For experimental procedures, each pen was supplied with a small nest box and a cardboard box (so cottontails could remove themselves from mallards), an elevated platform (so mallards could remove themselves from cottontails), a shallow pond, a poultry watering device (placed into the pen on day 1), a shallow food tray, a piece of sod, alfalfa, and an enrichment toy (Fig. 1). Food (MannaPro®, Pro Formula, St. Louis, MO; Game Bird Maintenance, Purina Mills, LLC, St. Louis, MO; feeds were placed in the same tray) and water were replenished each day as needed. Treated water (see above) was used throughout the experiment for both ponds and watering devices.

Ten commercially purchased mallards were utilized in the two studies. The mallards were bled prior to being moved into the study room to test for antibodies reactive with IAV.

### Transmission of IAV from striped skunks to mallards

Striped skunks were chemically anesthetized (intramuscular injection of a 5:1 ratio of ketamine/xylazine), and nasally inoculated with approximately 10^6^ EID_50_ of an avian IAV H4N6^15^ delivered in 1 mL of BA-1 viral transport media (see^7^). They were then locked in their nest boxes for two to three hours while they recovered from anesthesia.

**Figure 2:**
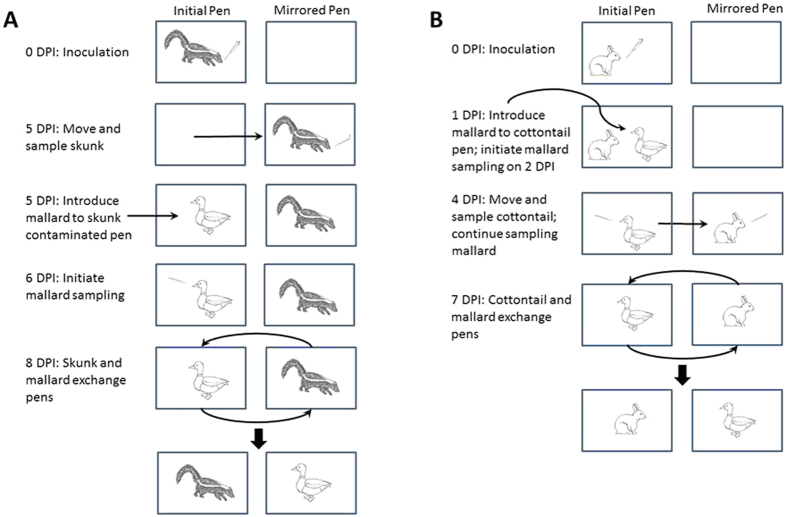
Experimental diagram of major steps used in interclass transmission studies with striped skunks (**A**) and cottontail rabbits (**B**) as virus donors to mallards. The term DPI refers to days post inoculation. The images were drawn by Elizabeth A. Draves.

Striped skunks were only sampled on a single occasion (5 DPI) during their course of infection to maximize the amount of shed virus that remained in the pens so that it was available to mallards. For sampling on 5 DPI, striped skunks were lightly chemically immobilized as described above and moved to mirrored pens on the other side of the same animal room. Following pen transfer, oral swab (stored in 1 mL of BA-1 diluent) and nasal flush (approximately 500 μl of BA-1 diluent utilized in each nostril) samples were collected. Samples were stored on ice packs and were subsequently transferred to −80 °C freezers immediately following the conclusion of processing. Striped skunks were detained in their nest boxes for 2–3 hours to recover from anesthesia. Mallards were introduced to the pens the skunks previously occupied just prior to the skunks being released from their nest boxes in their mirrored pens (Fig. 2A). In addition, a single mallard was placed in a pen that had not been occupied by a skunk to serve as an aerosol control. The paired skunks and mallards (with the exception of the control mallard) exchanged pens on 8 DPI (3 days post contact [DPC]), which corresponded to the peak shedding day of striped skunks during a previous study^6^. The mallards were sampled (fecal, oral, and cloacal swabs) on 1–15 DPC. During daily sampling, mallards were always sampled in the same order and prior to animal care of skunks in order to minimize the possibility of mechanical transmission of IAV from skunk pens to mallard pens during sampling and maintenance. In addition, a footbath was located outside of each pen to disinfect boots prior to working in another pen and gloves and gowns were changed for each pen. On 20 DPI, skunks were anesthetized with the methods mentioned above, bled, and euthanized with an injection of Beuthanasia-D Special (Schering-Plough Animal Health Corp., Union, NJ). On 15 DPC, mallards were bled and euthanized with the same methods used for skunks.

### Transmission of IAV from cottontail rabbits to mallards

After thoroughly sanitizing the structure, cottontails were moved into the same ABSL-2 building and pens as described above five days prior to the initiation of experiment 2 so they could acclimatize and explore their new pens. While under isoflurane anesthesia, cottontails were nasally inoculated with approximately 10^5.4^ EID_50_ of the same virus that was utilized for striped skunks delivered in 250 μl of BA-1. These animals were held in a different part of the animal room for approximately 4 hours after which they were moved to their respective pens. Cottontail rabbits have been shown to peak and clear their influenza infections more quickly compared to striped skunks^6^,^14^. Cottontails were co-housed with mallards for 3 days from 1–4 DPI, after which they were sampled (oral and nasal: see striped skunk methods above) and transferred to mirrored pens while mallards remained in their original pens (Fig. 2B). Cottontails and mallards switched pens on 7 DPI and were euthanized at 21 DPI (see methods above), at which time a blood sample was collected for serological analyses. The paired mallards were sampled (fecal, cloacal, and oral swabs) from 1–15 DPC. The mallards were euthanized at 20 DPC (see methods above) at which time a blood sample was collected.

### Laboratory analyses

Nasal flushes and oral swabs from skunks and cottontails, and oral, fecal, and cloacal swabs from mallards were tested by real-time RT-PCR. Briefly, viral RNA was extracted using MagMax-96 AI/ND Viral RNA Isolation Kits (Life Technologies). RNA extracts were tested in duplicate using primers and a probe specific for the influenza type A matrix gene as previously described^16^ using CFX96 Touch thermocyclers (BioRad). Thermocycler conditions were as follows: 50 °C for 10 min, 95 °C for 3 min, and 40 cycles of 95°C for 15 sec and 55 °C for 30 sec. Calibrated controls with known viral titers (10^2^ EID_50_/mL–10^5^ EID_50_/mL) were also analyzed to construct 4 point standard curves. Sample viral RNA quantities were extrapolated from the standard curves and are reported as PCR EID_50_ equivalents/mL. Positive samples were defined as those yielding a two-well positive amplification with a Cq value of  ≤38. Virus isolation was conducted on all nasal wash samples and select swab samples (e.g., an early and late time point in the course of the mallard infections) in SPF embryonated chicken eggs following published protocols^17^.

Pre-exposure and post-experiment serum samples were analyzed with standard AGID tests^18^,^19^ for cottontails and by ELISA with the FlockCheck^®^ Avian Influenza MultiS-Screen Antibody Test Kit (IDEXX Laboratories, Inc, Westbrook, ME) for striped skunks and mallards. Details on the use of these procedures in the species tested have been published elsewhere^6^,^14^,^20^.

## Results

### Viral shedding by striped skunks during experiment 1

**Figure 3:**
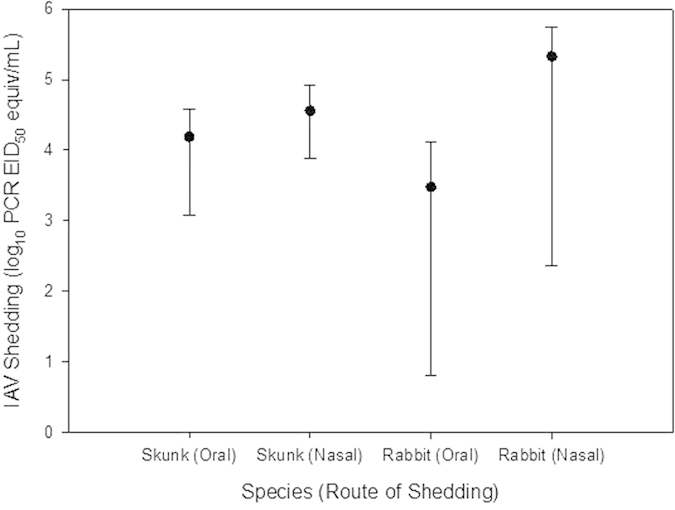
Means (dots) and ranges (vertical bars) for avian IAV shed nasally and orally on 5 DPI by inoculated skunks and on 4 DPI by inoculated cottontail rabbits.

All skunks yielded evidence of IAV infection at 5 DPI from oral swab and nasal flush samples (Fig. 3). Oral swab samples across all skunks averaged 10^4.19^ PCR EID_50_ equivalent/mL on 5 DPI (range = 10^3.08^ to 10^4.58^), while nasal wash samples averaged 10^4.57^ PCR EID_50_ equivalent/mL during the same day (range = 10^3.89^ to 10^4.92^). All tested nasal flush samples were confirmed positive for live virus by virus isolation. One skunk was observed sneezing on 6 DPI.

### Viral shedding by mallards during experiment 1

**Figure 4:**
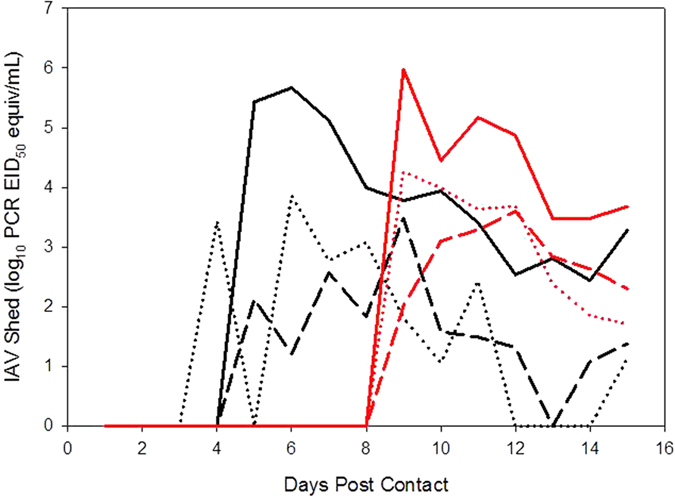
Shedding patterns of mallards that substantially shed avian IAV and were infected by indirect contact with infected striped skunks (black lines) and by direct or indirect contact with cottontail rabbits (red lines). Avian IAV shedding from feces is indicated by solid lines, from the oral route by dashed lines, and from the cloacal route by dotted lines.

One treatment mallard showed clear evidence of shedding and IAV infection (Fig. 4). In this individual, shedding was consistent with a previously published experimental infection study^21^. Shedding of viral RNA initiated on 4 DPC and continued through at least 15 DPC. Virus isolation was positive for all samples collected on 6 DPC (an early time point) but was negative for samples collected on 11 DPC (a late time point) when viral RNA concentrations were lower. Low levels of apparent shedding were observed in the three other treatment animals on one or more DPC. However, the apparent shedding in these animals was inconsistent among days and these animals failed to seroconvert during the study period. Thus, this fecal shedding may simply represent contamination of the pens by striped skunks or the ingestion and subsequent defecation of inactivated virus by mallards. In either scenario, it is clear that productive infections did not occur in these animals.

### Viral shedding by cottontail rabbits during experiment 2

All cottontails yielded evidence of IAV infection on 4 DPI from oral swab and nasal flush samples (Fig. 3). Oral swab samples across all cottontails averaged 10^3.47^ PCR EID_50_ equivalent/mL on 4 DPI (range = 10^0.8^ to 10^4.12^), while nasal flush samples averaged 10^5.33^ PCR EID_50_ equivalent/mL during the same day (range = 10^2.36^ to 10^5.74^). All nasal flush samples tested positive for live virus via virus isolation.

### Viral shedding by mallards during experiment 2

One treatment mallard showed clear evidence of shedding and infection (Fig. 4). Shedding initiated on 9 DPC and continued through at least 15 DPC. Two of three swab samples from this duck tested positive for live virus on both 9 and 15 DPC. Low levels of apparent shedding were observed in two other treatment animals on a single DPC. However, these animals failed to seroconvert during the study period and yielded evidence of viral RNA in a single fecal sample only. Thus, the positive fecal samples from these animals likely represent contamination of the pens by cottontails or ingestion and defecation of inactivated virus by mallards.

### Serology

All animals used in these experiments were determined to be antibody negative prior to the initiation of the experiments. Subsequently, all striped skunks and cottontail rabbits showed strong evidence of seroconversion at the end of the study period (20 and 21 DPI, respectively). A single mallard from experiment 1 and a single mallard from experiment 2 also yielded strong evidence of seroconversion at the end of the study period.

### Discussion and Conclusions

This study suggests that a common peridomestic mammal, the striped skunk, can successfully transmit IAV to mallards through indirect contact by contaminating shared food, water, and environmental resources. Of interest, the mallard that showed clear evidence of acquiring an IAV infection was paired with the skunk that shed the lowest quantities of viral RNA when compared to other skunks used in this experiment. The control mallard (to assess aerosol transmission) used in this experiment did not yield evidence of a productive infection. However, considering that only one-fourth of the mallards placed in treatment cages produced productive infections, this result is not surprising.

A single mallard became infected during the cottontail transmission study. As in the case of striped skunks, the cottontail that successfully transmitted IAV to a mallard was the animal that shed the lowest quantities of viral RNA as compared to the other cottontails. Thus, this transmission event suggests that transmission may have been affected by the behavior of the donor and recipient animals. Notably, cottontails were observed to more readily eat the duck diet as compared to rabbit maintenance diet.

No observations of cottontails interacting with mallards were noted when these animals were co-housed. In addition, the IAV positive mallard in experiment 2 (the cottontail rabbit study) started shedding after mallards and cottontail rabbits were no longer co-housed and after the animals had switched pens. Thus, both experiments appear to have relied upon environmental transmission of IAV to mallards, thereby indicating that the mallards received an undefinable dose of IAV, likely over multiple days. However, once infected the mallards yielded consistent shedding for several days. Overall, these data suggest that at least three items may be important to IAV transmission in the situations and environments outlined in this study. First, a mammal must acquire an IAV infection and must shed the virus, albeit these data suggest that animals that moderately shed the virus can successfully contaminate an environment for successful transmission to susceptible avian species. Second, the individual behavior of the donor animals may be a factor for shedding adequate virus into important facets of an environment, such as food and water sources, for successful transmission. Third, the bird acquiring an infection must ingest enough live virus to become infected. Thus, transmission may be highly dependent on where and when the donor animals shed the virus and individual birds use of pens and resources may enhance the potential of environmental transmission. The large pens utilized in both studies may have enhanced environmental transmission by not only accounting for species-specific habitat preferences (e.g., ducks and ponds/sod, rabbits and sod, skunks and den boxes), but also by producing overlap in areas of sustenance acquisition for both species (e.g., shared food and water devices). Alternatively, the large pens may have diluted transmission in some instances, as a smaller cage may have produced larger quantities of virus in small locations. Future studies should further evaluate the generality of the transmission events found in the current study. Moreover, studies that manipulate the size of shared environments could help to resolve the importance of pen size.

Certain wildlife species may become habituated to anthropogenically modified habitats, especially those associated with abundant food resources. Such behavior, at least in the context of multiple farms, could facilitate the movement of IAV from farm to farm if a mammal were to become infected at one farm and then travel to a second location^6^. This could be enhanced by the extended shedding of IAV previously noted in striped skunks^6^. In addition, it has been proposed that certain mammal species could circulate an IAV from a rural area, such as one containing waterfowl, to poultry-rearing facilities^5^. The current study provides evidence that select mammals can environmentally transmit IAV to mallards. In terms of high density poultry, it is quite possible that a single viral intrusion into the facility could subsequently transmit among a large number of poultry rapidly, dependent on the virus strain. As such, the potential intrusion of select peridomestic mammals into poultry facilities should be accounted for in biosecurity plans.

In recent years a greater emphasis has been placed on the potential roles of environmental transmission in IAV epidemiology^22^. For example, it has been suggested that a minor number of environmental transmission events may allow a virus to persist in small populations where primary transmission mechanisms are insufficient for viral maintenance^23^. However, this transmission mechanism may be greater for LP as compared to HP IAV^24^. Nonetheless, environments have been suggested as potential transmission facilitators among a diverse suite of hosts^25^. The results of this study suggest that certain mammals may contribute to IAV environmental transmission events in certain situations, although their contributions to these events are unknown at this time. Given that a fraction of both species tested successfully transmitted IAV to mallards, it is reasonable to assume that if these types of events were to occur in high-density poultry situations, a single incursion could provide an unfortunate spark.

## Additional Information

**How to cite this article**: Root, J. J. *et al.* When fur and feather occur together: interclass transmission of avian influenza A virus from mammals to birds through common resources. *Sci. Rep.* **5**, 14354; doi: 10.1038/srep14354 (2015).
